# Bromodomain-containing 4 as a therapeutic target in inflammatory bowel diseases and colorectal cancer

**DOI:** 10.3389/fphar.2025.1682223

**Published:** 2025-11-12

**Authors:** Irene Marafini, Rachele Frascatani, Marco Colella, Elena De Cristofaro, Giovanni Monteleone

**Affiliations:** 1 Gastroenterology Unit, Azienda Ospedaliera Policlinico Tor Vergata, Rome, Italy; 2 Department of Systems Medicine, University of “Tor Vergata”, Rome, Italy

**Keywords:** Crohn’s disease, ulcerative colitis, cytokines, mucosal immunity, colorectal cancer

## Abstract

Bromodomain-containing protein 4 (BRD4), a component of the bromodomain and extraterminal domain (BET) family, acts as a scaffold for transcription factors at promoters and super-enhancers, with the downstream effect of positively regulating the activity of signaling pathways, which sustain the inflammatory properties of immune cells and the expression of oncogens. These discoveries have boosted an intensive experimental work aimed at exploring the involvement of BRD4 in the pathogenesis of immune-mediated diseases and malignancies. As a result of these studies, there has been a considerable interest in the development of BRD4 inhibitors, which are now ready to be tested in clinical trials. In this article, we review the data about the expression and role of BRD4 in patients with Crohn’s disease and patients with ulcerative colitis, the major human inflammatory bowel diseases (IBD), as well as in patients with colorectal cancer (CRC). We also discuss the more recent data supporting the therapeutic benefit of BRD4 inhibitors in both IBD- and CRC-like mouse models.

## Introduction

BRD4 is a member of the Bromodomain and Extraterminal (BET) protein family, which also includes mammalian BRD2, BRD3, and the testis/ovary-specific BRDT. BRD4 functions as a scaffold for transcription factors at promoters and super-enhancers, and a histone acetyltransferase that acetylates histones H3 and H4, with the downstream effect of promoting the transcription of multiple genes (Devaiah et al., 2016). BRD4 contains two tandem bromodomains of 110 amino acids that structurally form 4 α-helices and 2 loops, which facilitate the interaction with acetylated lysine residues on target proteins, and an extraterminal domain that acts as an epigenetic “reader” (Dhalluin et al., 1999; Wu and Chiang, 2007). The two bromodomains of BET proteins have distinct functions in gene transcription. The second bromodomain of BRD4 is dedicated to interaction with acetylated histones H3 and H4, which leads to the recruitment of the ubiquitous positive transcription elongation factor b (P-TEFb), allowing *de novo* gene transcription, whereas the first one functions to anchor the activated BRD4/protein complex to target genes in chromatin through binding to acetylated histone H4 (Zhang et al., 2012; Schroder et al., 2012). BRD4 exists in 2 isoforms, long and short, which differ in their abundance in different cell types: the long isoform acts as a transcriptional coactivator, and the short isoform corresponds to an alternative splice variant (Wang N. et al., 2021).

**FIGURE 1:**
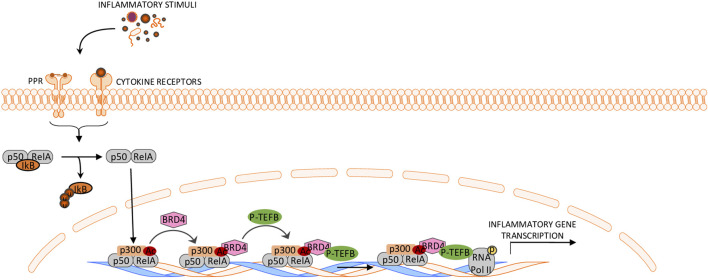
Schematic representation of BRD4 function in the control of the NF-κB pathway. The cytosolic p50/RelA dimer dissociates from IκB and translocates to the nucleus, where RelA is acetylated by CBP/P300. Acetylated RelA interacts with BRD4, which recruits P-TEFb, leading to phosphorylation of RNA polymerase II and activation of NF-κB-dependent transcription. This ultimately drives the transcription of pro-inflammatory genes.

BRD4 positively regulates the activity of transcription factors and/or signaling pathways, which sustain the inflammatory properties of immune cells. For instance, in macrophages, BRD4 interacts with the acetylated p65 subunit of NF-κB and increases its activity and stability in the nucleus (Hajmirza et al., 2018), thus resulting in enhanced transcription of pro-inflammatory NF-κB targets (Huang et al., 2009) (Figure 1). Consistently, BRD4 inhibitors disrupt BET protein-mediated activation of NF-κB, thereby attenuating the progression of several NF-kB-associated inflammatory diseases (Meng et al., 2014; Jahagirdar et al., 2017). Notably, not all the NF-κB target genes in specific cell populations are dependent on BET proteins for maximal transcriptional activation. Studies in pancreatic β cells showed that BRD4 inhibition leads to a marked suppression of genes mediating extracellular and inflammatory responses, while those involved in maintaining cell homeostasis and integrity are independent of BET proteins (Nord et al., 2025).

Additionally, BRD4 promotes the activation of dendritic cells, enhances the differentiation and/or function of effector CD4^+^ T cells, and interferes with the induction of counter-regulatory mechanisms (Remke et al., 2021; Sun et al., 2015; Cheung et al., 2017). BRD4 is a positive regulator of the MYC oncogene, the expression of which is deregulated in many cancers, and accumulating evidence supports the involvement of BRD4 in the progression of many malignancies (Loven et al., 2013).

In this article, we review the data about the expression of BRD4 in patients with inflammatory bowel diseases (IBDs) and patients with colorectal cancer (CRC) and discuss the more recent findings supporting the therapeutic benefit of BRD4 inhibitors in both IBD- and CRC-like mouse models.

## BRD4 amplifies inflammatory pathways in IBD

**FIGURE 2:**
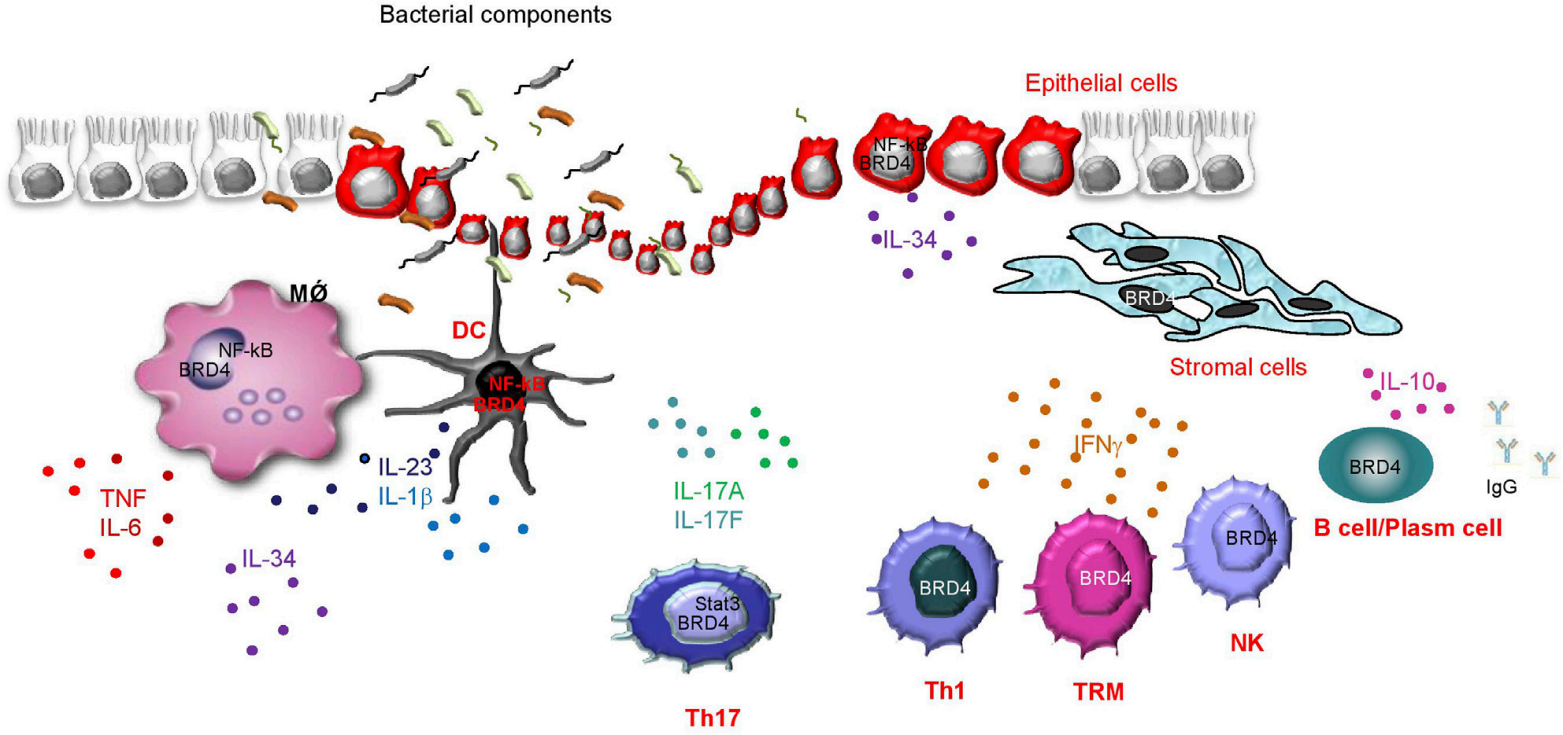
Schematic view illustrating which cells express BRD4 in the gut of patients with inflammatory bowel diseases (IBD), as well as some of the identified or presumed immune functions of BRD4, as supported by both studies with various BRD4 inhibitors using either mucosal cells isolated from IBD tissues or colons of mice with IBD-like colitis, and findings in other biological systems. BRD4 positively controls the activity of NF-kB in macrophages (MǾ), dendritic cells (DC), and epithelial cells, thereby enhancing the production of a vast array of effector cytokines. In Th17 cells, BRD4 controls Stat3 activity and enhances Th17-related cytokine synthesis (i.e., IL-17A and IL-17F). BRD4 also sustains IFN-g production in Th1 cells, memory T cells (TRM), and natural killer (NK) cells, even though the molecular mechanism by which BRD4 controls IFN-g synthesis in each cell type remains unknown. In IBD mucosa, CD19^+^ B cells express high levels of BRD4, and studies in other systems indicate a positive effect of BRD4 in sustaining B cell-derived IL-10 production as well as in human plasma cell differentiation and production of IgG.

Crohn’s disease (CD) and ulcerative colitis (UC), the two major IBDs in human beings, are chronic inflammatory disorders of the gastrointestinal tract of unknown etiology (Dolinger et al., 2024). In both CD and UC, the tissue-damaging pathological process is driven by an excessive immune response against microbial antigens, which is poorly regulated by counter-regulatory mechanisms (Monteleone et al., 2023). The active phases of IBD are marked by a mucosal infiltration with several innate and adaptive immune cells, which secrete huge amounts of inflammatory cytokines targeting both immune and non-immune cells (e.g., stromal cells, epithelial cells), thus contributing to expanding the detrimental inflammatory response and promoting mucosal damage (Monteleone et al., 2023). Notably, mucosal cells in IBDs express high levels of p300/CBP (Monteleone et al., 2005), an acetyltransferase that selectively promotes BRD4 recruitment at enhancers (Narita et al., 2021). These observations and the demonstration that BRD4 is a positive regulator of NF-kB, the activity of which is up-regulated in IBD mucosal cells (Neurath et al., 1996), prompted us to explore the expression and function of BRD4 in IBDs. By using mucosal biopsy samples taken from IBD patients and controls, we initially showed that BRD4 expression was more pronounced in IBD tissue than in the unaffected gut mucosa, and this was evident at both RNA and protein levels. In IBDs, BRD4 content was increased only in the mucosal areas with endoscopic lesions, arguing for a role of the IBD-associated active inflammation in the induction of BRD4 (Franze et al., 2024a). This hypothesis is further supported by the demonstration that BRD4 expression was not up-regulated in duodenal biopsy samples of patients with active celiac disease, a gluten-dependent chronic inflammatory disease of the small intestine. The induction of BRD4 in IBD tissue seems to be highly selective, as no increase was seen in the RNA expression of BRD2 and BRD3 (Franze et al., 2024a). In line with previous studies showing a diffuse expression of BRD4 in immune and non-immune cells (Wang N. et al., 2021), we showed that virtually all the immune cell types in the gut were positive for BRD4 and that, in IBD mucosa, T lymphocytes and antigen-presenting cells were the major sources (Franze et al., 2024a) (Figure 2). To assess the functional role of BRD4 in IBD-related immune response, we inhibited BRD4 in IBD mucosal cells with a specific antisense oligonucleotide and then assessed the production of inflammatory cytokines. BRD4 inhibition was followed by a significant downregulation of the synthesis of TNF, IFN-γ, and interleukin (IL)-17A, cytokines that are overproduced in the active phases of the disease (Marafini et al., 2019). We also documented enhanced expression of BRD4 in the inflamed gut of mice with experimental colitis, and showed that BRD4 content paralleled the overexpression of inflammatory cytokines (Franze et al., 2024a). To further prove that BRD4 drives pathogenic signals in the gut, we intraperitoneally injected colitic mice with the small-molecule BET inhibitor JQ1, which masks bromodomain acetyl-lysine-binding pockets (Zhou et al., 2020). JQ1 treatment down-regulated the inflammatory cytokine response and attenuated the ongoing colitis (Franze et al., 2024a).

Along the same line is the demonstration that inhibition of the first bromodomain of BET proteins with the small molecule MS402 blocks mainly T helper (Th)-17 cell differentiation over Th1, Th2, or T regulatory cells (Tregs), thereby preventing and ameliorating T-cell transfer-induced colitis in mice (Cheung et al., 2017). The preferential MS402-mediated inhibition of Th17 cell differentiation is supported by the inhibitory effect of the compound on the transcriptional activation of the specific Th17 cell-associated transcription factor, Rorc in Th17 cells, over the suppression of transcription factors associated with Th1 (i.e., tbx21), Th2 (i.e., gata3), and Tregs (i.e., foxp3) differentiation (Snyder et al., 2021). Mechanistically, MS402 treatment leads to a marked reduction of BRD4 and Cdk9 occupancy and RNA Polymerase II (RNA PolII) Ser2 phosphorylation level at the Stat3 binding sites in IL-17A/F and Rorc loci, which is required for transcription elongation (Zhang et al., 2012). More recently, Jeong and colleagues showed that KB-0118, another BET bromodomain inhibitor targeting BRD4, inhibited TNF, IL-1β, and IL-23 production, and selectively suppressed Th17 cell differentiation *in vitro*. In mice with either dextran-sulfate sodium (DSS)-induced colitis or T cell transfer-mediated colitis, KB-0118 attenuated disease severity and lowered IL-17 expression (Jeong et al., 2025). However, in these studies, the drug was given to mice simultaneously with the induction of colitis, thus leaving open the question of whether it is able to reverse the already established mucosal inflammation. Ma and colleagues recently showed that BRD4 is aberrantly activated in the inflamed colon of IBD patients and ZL0516, an oral BRD4 BD1 inhibitor, reduced TNFα- and LPS-induced production of inflammatory cytokines in human colonic epithelial cells and peripheral blood mononuclear cells, and both prevented and cured experimental colitides in mice as a result of its ability to block the activation of the BRD4/NF-κB signaling pathway (Ma et al., 2025).

We also showed that BRD4 positively controls the expression of IL-34, another cytokine that is supposed to amplify detrimental signals in IBD mucosa. Specifically, we documented that, in the inflamed tissue of IBD patients, IL-34 and BRD4 co-localize in both lamina propria mononuclear cells (LPMCs) and epithelial cells, and knockdown of BRD4 in IBD LPMCs with a specific antisense oligonucleotide reduced IL-34 production. Similar results were documented in the colons of mice with DSS-induced colitis treated with JQ1 (Franze et al., 2024b).

Altogether, these findings indicate that BRD4 is highly expressed in IBDs and suggest that BRD4 inhibition could help dampen pathological gut inflammation.

Several advanced therapies are currently approved for the treatment of CD and UC, including biologics targeting TNF (infliximab, adalimumab), IL-12/23p40 (ustekinumab), IL-23p19 (Risankizumab, mirikizumab, guselkumab), integrins (vedolizumab), and small molecules inhibiting Janus kinase pathways (tofacitinib, upadacitinib, filgotinib) and sphingosine 1 phosphate (ozanimod, etrasimod). Although these agents can induce clinical and endoscopic remission in a proportion of patients, up to 40–50% experience primary non-response or secondary loss of response over time, in part due to cytokine redundancy and convergent signaling pathways. Given that BRD4 acts upstream of several inflammatory transcriptional programs, including NF-κB, its inhibition could theoretically suppress multiple cytokine networks simultaneously, potentially overcoming the limitations of single-pathway blockade (Ma et al., 2023; Ma et al., 2024). Importantly, no BRD4-specific inhibitors have yet been approved for clinical use in IBD, and all current data derive from preclinical models. Therefore, further studies are needed to establish their selectivity, mucosal healing potential, and long-term safety compared with existing immunomodulators.

## The role of BRD4 in colorectal cancer

CRC is one of the most common and fatal cancers worldwide (Bray et al., 2018). The majority of CRC arises as a sporadic disease, while in a small percentage of cases (2–3%), CRC arises in patients with extensive and long-standing IBD (colitis-associated cancer, CAC) (Francescone et al., 2015; Rutter et al., 2020). Several somatic and germline mutations are supposed to drive sporadic CRC at the molecular level and can be linked to well-defined disease stages of tumor progression. Based upon the initiating molecular alterations, CRC can be divided into 3 main subtypes: 1. Nearly 60% of CRC arise from the chromosomal instability (CIN) pathway and are distinguished by aneuploidy and recurrent chromosomal amplifications at distinct genomic loci; 2. CpG island methylator phenotype (CIMP), which comprises 20% of CRC and is characterized by poor patient outcomes; 3. microsatellite instability (MSI), a hypermutable phenotype caused by the loss of DNA mismatch repair activity, accounting for nearly 15% of CRC (Issa, 2008; Ogino and Goel, 2008).

Although mutations of various tumor-suppressor genes (e.g., APC, KRAS, p53) and oncogenes (e.g., cMYC) have been described in a high percentage of CRC (Dulak et al., 2012) (Powell et al., 1992), epigenetic and genetic alterations are likely to act synergistically in CRC development (Hammoud et al., 2013; Ying and Tao, 2009; Suzuki et al., 2004). Activation of oncogenes is partly mediated by superenhancers, which are segments of DNA typically occupied by multiple transcription factors and recruiting coactivators and RNA Pol II to target genes (Ong and Corces, 2011). BRD4 is recruited to superenhancers and consequently functions as an epigenetic reader to promote transcription of superenhancer-marked genes in cancer cells (Dey et al., 2000). Numerous researchers have investigated the role of BRD4 in CRC. To assess the epigenetic regulation of BRD4 in CRC, Rodriguez and colleagues used bisulfite pyrosequencing to determine the methylation status of a CpG cluster located 607 bp from the BRD4 transcription start site in healthy colon epithelium and CRC cell lines (Rodriguez et al., 2012). The authors showed that BRD4 is epigenetically downregulated in human CRC. They also showed that restoring BRD4 expression in CRC cell lines did not affect cell proliferation or clonogenic potential *in vitro*, but reduced the tumor growth after xenograft injection, thus leaving unexplained the link between BRD4 repression and CRC. In contrast, in an elegant study aimed at uncovering the key epigenetic regulators that promote CRC cell growth, McCleland and colleagues showed that inhibition of BRD4 by JQ1 reduced MYC expression in cancer cells and led to a preferential reduction of the growth of a subset of epigenetically dysregulated CIMP-positive CRC. By transcriptomic and genomic analyses of CIMP-positive CRC tissues, the authors identified a distinct superenhancer regulating cMYC transcription. Such a superenhancer transcribes the long noncoding RNA colon cancer-associated transcript 1 (CCAT1), which is sensitive to BET inhibition (McCleland et al., 2016). In CRC, superenhancers are enriched not only for BRD4 but also for other proteins, such as the coactivator Mediator, which cooperates with BRD4 in sustaining the expression of cancer-acquired superenhancer genes (e.g., MYC) (Kuuluvainen et al., 2018). Inhibition of BRD4 in CRC cells triggers endoplasmic reticulum stress and enhances the transcriptional induction of death receptor 5 (DR5), a key component of the extrinsic apoptotic pathway, which promotes the chemosensitization and apoptotic effects of BET inhibitors (Tan et al., 2019). Similar anti-neoplastic effects have been seen following *in vitro* and *in vivo* treatment of CRC cells with SF1126, a BRD4 and phosphatidylinositol 3-kinase dual inhibitor, which activates p38 signaling thus inducing cytotoxicity and apoptosis (Qin et al., 2019), a combined treatment with AZD5153, a BRD4 inhibitor, and the PARP inhibitor BMN673 (Zhang et al., 2019) or the nontoxic dietary supplement, docosahexaenoic acid, and JQ1 (Ding et al., 2020).

Remoting of acetyl groups from lysine residues of histones by histone deacetylases (HDACs) can affect chromatin conformation and inhibit gene expression and the biological functions of some transcription factors. Consistently, dysfunction of HDACs has been linked to the development of tumors (Barneda-Zahonero and Parra, 2012). Treatment of CRC cells with romidepsin (FK228), an HDAC inhibitor, induces G0/G1 cell cycle arrest, thereby inhibiting proliferation and increasing apoptosis. The antineoplastic effects of romidepsin are accompanied by enhanced PD-L1 expression in CRC cells through a process that relies partly on BRD4. Administration of romidepsin to both mice with subcutaneous transplant CRC-derived tumors and mice with CAC increases the percentage of FOXP3+ regulatory T cells, and decreases the ratio of Th1/Th2 cells and IFN-γ secretion by CD8^+^ T cells, while combination with an anti-PD-1 antibody reverses these effects and reduces tumor growth (Shi et al., 2021). Chemoresistance of CRC cells is also largely influenced by tumor cell heterogeneity and the immunosuppressive tumor microenvironment (Bayik and Lathia, 2021). Notably, expression of BRD4 in tumor-associated type 2 macrophages is crucial in promoting their persistence with the tumor microenvironment and conferring resistance of CRC cells to oxaliplatin, a finding that seems to rely on the ability of BRD4 to enhance the transcription of SERPINE1 gene and, hence, production of plasminogen activator inhibitor-1 (PAI-1) (Pan et al., 2025), a well-known promoter of tumorigenesis (Placencio and DeClerck, 2015). The clinical relevance of such observations is supported by the demonstration that the levels of BRD4 in tumor-associated macrophages and PAI-1 in tumors are elevated in CRC patients with chemoresistance and correlate with shorter recurrence-free survival (Pan et al., 2025).

**FIGURE 3:**
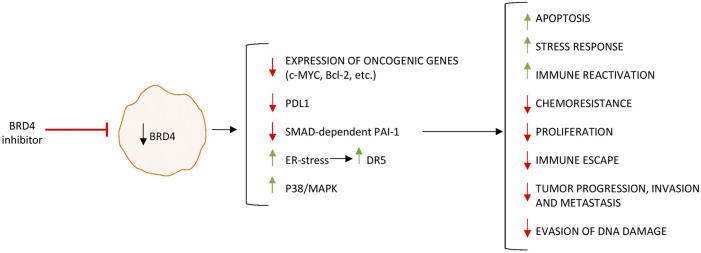
BRD4 inhibition regulates the expression of genes involved in tumor growth, survival, and immune evasion, leading to decreased proliferation and impaired tumor-promoting functions in colorectal cancer cells. Upward arrows (↑) indicate upregulation, whereas downward arrows (↓) indicate downregulation of gene/protein expression.

Altogether, the above data indicate that BRD4 sustains several pathways that ultimately promote colon tumorigenesis (Figure 3). Conventional treatment of CRC includes surgical resection, chemotherapy, and targeted therapies such as anti-EGFR antibodies and anti-VEGF agents, while immune checkpoint inhibitors are reserved for microsatellite instability-high tumors. Despite these options, resistance and recurrence remain major challenges, especially in microsatellite-stable and chemoresistant tumors (Steup et al., 2025; Yu et al., 2024).

Epigenetic dysregulation contributes to such resistance, suggesting that targeting chromatin readers like BRD4 could represent a novel complementary strategy. BRD4 inhibition or degradation has shown synergistic activity with chemotherapy and immune checkpoint blockade in preclinical CRC models, supporting the view that BRD4 targeting might re-sensitize resistant tumors and modulate the immunosuppressive microenvironment. Nevertheless, the efficacy and safety of these agents in humans remain to be defined.

For a complete list of synthesized BRD4 inhibitors and their chemical structures, the reader is referred to recently published reviews (Ma et al., 2023; Ma et al., 2024).

## Discussion

The findings described in this article support the involvement of BRD4 in the pathogenesis of IBDs and CRC, given that the expression of BRD4 is up-regulated in the affected tissues of those patients, and inhibition of BRD4 expression/function attenuates the pathological process in murine models of IBDs and CRC (Table 1). Collectively, these findings raise the possibility that BRD4 blockers could enter into the future armamentarium of patients with IBD and patients with CRC. However, several issues need to be explored before launching BRD4-targeted therapy into clinical trials. The data accumulated in IBD patients indicate that BRD4 is overexpressed by immune and non-immune cells (e.g., epithelial cells). Since BRD4 controls NF-kB, the activation of which in gut epithelial cells seems to elicit protective rather than detrimental effects (Neurath et al., 1998), it is conceivable that suppression of BRD4 in gut epithelial cells could somehow impair the healing process of mucosal damage. Future studies are needed to better investigate the impact of BRD4 inhibition on additional transcriptional factors and signaling pathways (e.g., Stat3), which are supposed to influence gut immune homeostasis (Pooladanda et al., 2021; Wang W. et al., 2021). The available data indicate that antisense oligonucleotide-induced knockdown of BRD4 in IBD immune cells leads to a downregulation in the production of IFN-γ, IL-17A, and TNF (Franze et al., 2024a). Since such cytokines are produced by several immune cell types (e.g., polarized CD4+Th cells, memory T cells, CD8^+^ T cells, innate lymphoid cells, MAIT cells), it remains unclear whether BRD4 controls the function of single or multiple cell populations. Moreover, it is unknown whether BRD4 regulates counter-regulatory mechanisms, which are defective in IBD mucosa (Maresca et al., 2025; Caruso et al., 2009). In this context, it is noteworthy that BRD4 is expressed in CD19^+^ B cells infiltrating the affected IBD mucosa (Franze et al., 2024a), and studies in murine splenic and peritoneal B cells showed that JQ1 inhibited IL-10 secretion, inferring the involvement of BRD4 in the function of IL-10-producing regulatory B cells (Lee et al., 2017). Moreover, BRD4 regulates human plasma cell differentiation and production of IgG (Zeng et al., 2022), even though its involvement in the IBD-associated humoral response remains to be ascertained.

**TABLE 1:** Summarizing the chemical structure and the effects of the BRD4 inhibitors described in the text.

Inhibitor’s name	Chemical structure	*In vitro*/*in vivo* effects	References
JQ1	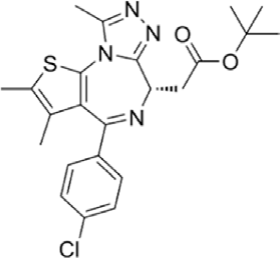	JQ1 down-regulates inflammatory cytokines and attenuates colitis JQ1 reduces MYC expression in cancer cells	Franze et al. (2024a), Zhou et al. (2020), Franze et al. (2024b), McCleland et al. (2016), Ding et al. (2020)
MS402	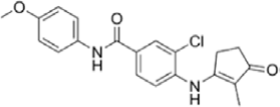	MS402 blocks Th-17 cell differentiation over Th1, Th2, or Tregs, thereby preventing and ameliorating T-cell transfer-induced colitis in mice	Cheung et al. (2017)
KB-0118	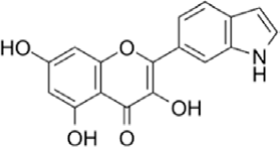	KB-0118 inhibits TNF, IL-1β, and IL-23 production, and selectively suppresses Th17 cell differentiation *in vitro*; *in vivo* attenuates DSS-induced colitis	Jeong et al. (2025)
ZL0516	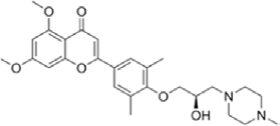	ZL0516 reduces TNFα- and LPS-induced production of inflammatory cytokines *in vitro*, and prevents and cures experimental colitis in mice	Ma et al. (2025)
SF1126	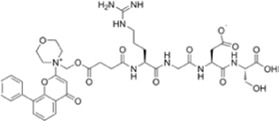	Antineoplastic effect: SF1126 activates p38 signaling thus inducing cytotoxicity and apoptosis	Qin et al. (2019)
AZD5153	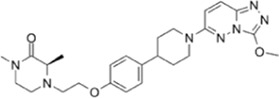	Antineoplastic effect: AZD5153 activates p38 signaling thus inducing cytotoxicity and apoptosis	Zhang et al. (2019)
A1874	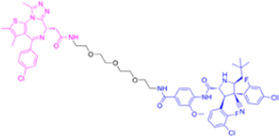	A1874 inhibits cell viability, proliferation, migration, and invasion of primary CRC cells and established CRC cell lines	Tan et al. (2018)
dBET1	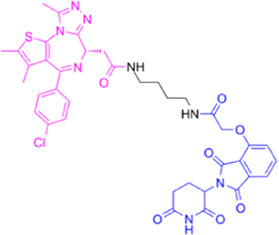	dBET1 reduces CRC cell growth, and decreases chemoresistance and immune resistance of cancer	Hines et al. (2019)
dBET6	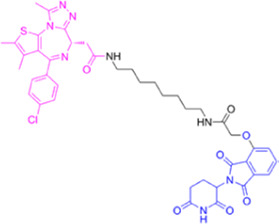	dBET6 reduces CRC cell growth, and decreases chemoresistance and immune resistance of cancer	Winter et al. (2015)

CRC, colorectal cancer; Th, T helper; Tregs, T regulatory cells; TNF, tumor necrosis factor; IL, interleukin; DSS, dextran sulphate sodium; LPS, lipopolysaccharide.

Little is known about the role of BRD4 in the induction and progression of fibrogenesis, a phenomenon frequently seen in IBDs, particularly in CD. Studies investigating the activation process of hepatic stellate cells, a key step in liver fibrogenesis, have shown that the pro-fibrotic transforming growth factor (TGF)-β1 activates Stat3, thereby promoting BRD4 expression, and BRD4 knockdown interrupts the positive effects of Stat3 on the fibrogenic process induced by TGFβ1 (Xu et al., 2023). In this context, our immunostaining of IBD mucosal sections revealed that BRD4 is expressed not only by immune cells (i.e., CD3^+^ T cells, CD19^+^ B cells, CD68^+^ macrophages, and CD11c+DC) but also by stromal cells, suggesting a possible involvement of BRD4 in the function of such cells (Franze et al., 2024a). Finally, more functional studies would be needed to assess whether BRD4 blockers could potentiate the therapeutic effects of already approved targeted therapies in IBD. Similarly, more research is needed to assess at which stage of colon carcinogenesis BRD4 is induced and the prognostic value of BRD4 content. BRD4 is believed to be a promising anti-cancer drug target due to its strong positive effects on the expression of the transcription factor MYC, which is a well-known oncogenic master regulator and a driver of colon tumorigenesis (Dhanasekaran et al., 2022). However, given the heterogeneity of CRC, the candidates who are likely to benefit from BRD4 inhibitors remain to be defined.

So far, small-molecule inhibitors of BRD4 (e.g., JQ1) have shown promising anticancer effects in experimental models of CRC. However, in contrast to hematologic malignancies in which the BRD4 inhibitor-mediated downregulation of MYC correlates well with reduced cell proliferation, in solid tumors, the reduction of MYC expression and cell growth is less striking after single drug treatment (Delmore et al., 2011; Tan et al., 2018), suggesting the necessity for more effective compounds. Another approach to block BRD4 function in cancer cells is represented by the use of “PROteolysis-TArgeting Chimeras”, or “PROTACs” (Hines et al., 2019). PROTACs are heterobifunctional molecules that tether together a ligand that binds the protein of interest with another ligand that engages an E3 ubiquitin ligase, resulting in ubiquitination of the former and its subsequent degradation by the 26S proteasome. A novel BRD4-degrading PROTAC is A1874, which was shown to inhibit cell viability, proliferation, migration, and invasion of primary CRC cells and established CRC cell lines. In these cells, the A1874-induced degradation of BRD4 protein was accompanied by a downregulation of BRD4-dependent genes (i.e., MYC, Bcl-2, and cyclin D1) as well as by p53 protein stabilization and enhanced production of reactive oxygen species, the latter being also involved in the A1874-induced cell death and apoptosis (Qin et al., 2020). This suggests that the anti-tumoral effects of A1874 are mediated by both BRD4-dependent and -independent mechanisms. Another BRD4 degrader is dBET1, a composite molecule consisting of JQ1 and a thalidomide moiety, which targets cereblon, a component of the ubiquitin ligase complex, thus facilitating the ubiquitination and proteasomal disintegration of the BET protein (Winter et al., 2015). Structural modification of dBET1 yielded an optimized compound, termed dBET6, which differs from dBET1 by an extended carbohydrate bridge (Winter et al., 2017). dBET6 appears to be more effective than dBET1 and JQ1 in reducing CRC cell growth, and decreases chemoresistance and immune resistance of cancer (Bauer et al., 2021).

Ongoing studies are also assessing the advantage of combined therapy of BRD4 inhibitors and Stat3 blockers or BRD4 inhibitors with chemotherapy over monotherapy in suppressing cancer growth (Ramadoss and Mahadevan, 2018; Duan et al., 2023). Moreover, there is intense research aimed at investigating the regulatory effects of specific miRNAs on the expression and function of BRD4 (Gen et al., 2021).

Another emerging challenge in the development of BRD4-targeted therapies lies in identifying the specific disease contexts in which BRD4 acts as a driver rather than a bystander of inflammation or tumorigenesis. In IBD and CRC, inter-individual variability in BRD4 expression, post-translational modifications, and interaction networks suggests that patient stratification will be essential to define responders to BET inhibition. In this perspective, integrating BRD4-related molecular signatures into multi-omic and single-cell profiling studies could guide precision approaches and unveil novel therapeutic windows. Such translational efforts may help reposition BRD4 modulation not as a universal target, but as a context-dependent strategy within the evolving landscape of epigenetic therapy.

Finally, whatever the indication of BRD4 inhibitors, further and appropriate long-term studies in murine models of gut inflammation and CRC are needed to define any possible adverse effects related to their use.
